# IntraVenous vs IntraOsseous access in Cardiac Arrest: the omitted economic question (IVOCA study)

**DOI:** 10.1016/j.resplu.2026.101305

**Published:** 2026-03-27

**Authors:** Alexis Marouk, Jean-Marc Agostinucci, François-Pierre Auffredou, Tomislav Petrovic, Jacques Metzger, Philippe Bertrand, Priscilia Hsing, Anne-Laure Feral-Pierssens, Frédéric Lapostolle

**Affiliations:** a AP-HP, Hôpital Avicenne, Service d’Urgences – SAMU 93, Bobigny 93000, France; b Université Paris Cité, Université Sorbonne Paris Nord, Inserm, UMR 1137 IAME, Paris 75018, France; c Université Sorbonne Paris Nord, LEPS UR 3412, Bobigny 93000, France; d Université Sorbonne Paris Nord, Inserm U942 MASCOT, AP-HP, Hôpital Avicenne, SAMU 93 – UF Recherche-Enseignement-Qualité, Bobigny 93000, France

**Keywords:** Intraosseous, Intravenous, Cardiac arrest, Cost, Cost-minimization, Resuscitation

## Abstract

**Aims:**

Intraosseous (IO) access is increasingly used during out-of-hospital cardiac arrest (OHCA) despite higher device costs and no proven clinical superiority over peripheral intravenous (IV) access. We conducted a cost-minimization analysis to estimate device-related costs under observed current practice and hypothetical alternative IV/IO strategies.

**Methods:**

Retrospective cohort study using regional data from the French national OHCA registry (RéAC), 2013–2024 (Seine-Saint-Denis, France). Patients receiving adrenaline (epinephrine) via IV or IO were included. Unit costs were €0.6 per IV catheter and €109 per IO needle. Scenario modeling was used to estimate device costs for alternative strategies (first-line IO versus stepwise IV-to-IO), using cumulative IV success rates (65% after one attempt to 99% after four), assuming 100% IO success, and applied to median annual registry volumes.

**Results:**

Among 10,737 OHCAs, 5350 (50%) patients were included (median age 62 years; 30% women). Access was IV only in 4128 (77%), IO only in 1092 (20%), and both in 130 (2%). Annual IO device costs increased from €5450 in 2013 to a peak of €16,350 in 2022, totaling €133,198 versus €2555 for IV. Return of spontaneous circulation (ROSC) occurred in 31% of cases (−0.3% annual decline); 30-day survival was 2.8% (−0.08% annual decline). Scenario modeling indicated that systematic first-line IO would correspond to €349 per ROSC and €3974 per survivor in device costs, whereas a strategy without IO would correspond to €2 per ROSC and €22 per survivor.

**Conclusion:**

Over the study period, IO use increased and generated substantially higher device costs. In the absence of proven clinical superiority, an IV-first strategy with selective IO use appears economically preferable.

## Introduction

Intraosseous (IO) access has been increasingly adopted during out-of-hospital cardiac arrest (OHCA) resuscitation, representing up to one-third of vascular access attempts.^1^ However, its broad implementation is subject to debate. Multiple studies showed that a peripheral intravenous (IV) line can be established in most patients within just two or three attempts.^2^, ^3^ While IO access may be faster and has higher rates of first-attempt success in some settings,^4^ large randomized controlled trials (RCTs) directly comparing IO-first vs IV-first access strategies failed to show a significant benefit in 30-day survival, sustained return of spontaneous circulation (ROSC) and favorable neurological outcome.^5^, ^6^

Despite this clinical equipoise, the economic implications of IO use have been largely neglected. IO devices are substantially more expensive, often exceeding €100 per needle compared with only a few cents for an IV catheter. Previous analyses have compared IO with central venous catheters in the hospital setting, suggesting that IO may be less costly than central lines, but such findings cannot be extrapolated to cardiac arrest, where IV access usually is readily available and inexpensive.^7^

Given the absence of proven clinical superiority of IO over IV access, we conducted a cost-minimization analysis to quantify device-related costs associated with current practice and to estimate the expected costs of alternative vascular access strategies in OHCA.

## Methods

### Study design and setting

We conducted a retrospective cohort study using regional data from the French national OHCA registry (RéAC), which prospectively records all OHCAs managed by the emergency medical services in participating centers.^8^ In France, prehospital advanced life support is delivered by physician-staffed mobile intensive care units (MICUs). Seine-Saint-Denis, a densely populated department northeast of Paris with ∼1.6 million inhabitants, has been consistently involved in the registry and served as the study setting.^9^ This study was conducted in accordance with the Declaration of Helsinki. The national registry RéAC had an authorization of the French National Data Protection Commission (CNIL authorization no. 910946 on 17/08/2015), which covers the secondary use of anonymized patient data for research purposes.

### Participants

All consecutive patients with OHCA between January 2013 and December 2024 were eligible. Patients were included if they underwent attempted advanced life support and received adrenaline (epinephrine). Patients were excluded if vascular access was achieved through routes other than peripheral IV or IO (i.e. central venous or endotracheal) or if access or data on vascular access or outcomes were missing.

### Data collection

Demographic data (age, sex), arrest circumstances (location, cause), early resuscitation characteristics (low-flow duration, bystander-initiated CPR, automated external defibrillator attachment and shock before MICU arrival, rhythm at MICU arrival), time from emergency call to first adrenaline administration, vascular access route (IV, IO, or both), and outcomes (ROSC with a palpable pulse for at least 1 minute and 30-day survival) were extracted from the registry. Data were collected prospectively by prehospital physicians using standardized forms and definitions (Utstein-style), and uploaded into the RéAC electronic database, as described in prior methodological reports.^10^

### Outcomes

The primary outcome was the economic cost of vascular access. Costs were analyzed over time and expressed overall and per clinical outcome (ROSC and 30-day survival). Unit costs were based on official 2024 prices from the Paris university hospital system (Assistance Publique–Hôpitaux de Paris): €0.6 for a standard 18G IV catheter and €109 for a 45-mm yellow IO needle (Teleflex®), without inflation adjustment. Ancillary materials common to both strategies (compresses, disinfectants, fixation) were not included. The cost of the driver device (EZ-IO® from Teleflex®; ∼€330) was also excluded.

To model cost-minimization, we considered strategies ranging from systematic first-line IO use to IO use after one to four failed IV attempts, and a strategy in which no IO cost was assigned. IV success rates were taken from the IVIO trial and modeled as cumulative probabilities: 65% after one IV attempt, 80% after two attempts, 90% after three attempts, and 99% after four attempts.^6^ Thus, in each scenario, the proportion of patients requiring IO corresponded to the complement of cumulative IV success at the specified step. IO insertion was assumed to be successful at the first attempt. For each strategy, we calculated annual device cost, cost per ROSC, and cost per 30-day survivor.

### Statistical analysis

Continuous variables were described as medians with interquartile ranges (IQR) and categorical variables were presented as counts and percentages. No statistical testing was performed between vascular access groups, as the objective was descriptive. Temporal trends (2013–2024) in costs and outcomes were analyzed using linear regression for ROSC and a logarithmic regression for IO cost, reported with the coefficient of determination (*R*^2^). Scenario-based economic modeling was conducted by calculating, for each study year, the annual numbers of included OHCAs having received adrenaline, ROSC, and 30-day survivors, and then using the median of these annual values to represent a typical study year. Statistical analyses were conducted using R software version 4.4.0 (R Foundation for Statistical Computing).

## Results

Between January 1, 2013, and December 31, 2024, 10,737 patients were recorded in the registry, of whom 5350 (50%) were included in the study. We excluded patients who did not receive adrenaline (*n* = 5327; 50%), then those who achieved vascular access through other routes than IV or IO (*n* = 58; 0.5%) and those with missing data on vascular access or outcomes (*n* = 2, <0.1%). Among included patients (*n* = 5350), 1625 (30%) were female and 3725 (70%) were male, with a median age of 62 years (IQR: 49–73), and 113 patients (2.1%) younger than 15 years. Cardiac arrest was of medical origin in 4511 cases (84%), and non-medical in 839 cases (16%) due to traumatic causes, overdose, drowning, electrocution, or asphyxia. The vascular access route was IV only in 4128 (77%) patients, IO only in 1092 (20%), and both IV and IO in 130 (2%) (Table 1).

**Table 1:** **Study population’s general characteristics and outcomes.** Data are presented as median (interquartile range) or numbers (percentage). AED, automated external defibrillator; CPR, cardiopulmonary resuscitation, IO, intraosseous; IV, peripheral intravenous; MICU, mobile intensive care unit; ROSC, return of spontaneous circulation.

	**Overall** ***N* = 5350**	**IV only** ***N* = 4128 (77)**	**IO only** ***N* = 1092 (20)**	**IV + IO** ***N* = 130 (2)**
Age (years)	62 [49–73]	63 [51–74]	58 [43–70]	57 [41–68]
Female sex	1625 (30)	1215 (29)	370 (34)	40 (31)
**Cause of cardiac arrest**				
Medical	4511 (84)	3534 (86)	876 (80)	101 (78)
Traumatic	364 (6.8)	227 (5.5)	122 (11.2)	15 (11.5)
Other^a^	475 (8.9)	367 (8.9)	94 (8.6)	14 (10.8)
Cardiac arrest located at home	3673 (69)	2861 (69)	736 (67)	76 (58)
Low flow duration (min)	40 [30–53]	40 [29–53]	42 [31–54]	41 [27–56]
Bystander CPR performed	3048 (57)	2368 (57)	589 (54)	91 (70)
AED attached before MICU arrival	4677 (87.4)	3618 (87.6)	949 (86.9)	110 (84.6)
AED shock before MICU arrival	1035 (19)	843 (20)	168 (15)	24 (18)
**Rhythm at MICU arrival**^b^				
Shockable	469 (8.8)	389 (9.4)	64 (5.9)	16 (12.3)
Non-shockable	4671 (87)	3557 (86)	1004 (92)	110 (85)
Spontaneous activity	210 (3.9)	182 (4.4)	24 (2.2)	4 (3.1)
Time from emergency call to adrenaline administration (min) c	25 [19–33]	25 [18–32]	25 [19–34]	24 [19–30]
ROSC with palpable pulse ≥1 min	1651 (31)	1324 (32)	257 (24)	70 (54)
30-day survival	151 (2.8)	131 (3.2)	13 (1.2)	7 (5.4)

a Including overdose, drowning, electrocution, or asphyxia.

b Missing data for 23 patients were considered as non-shockable rhythm.

c Missing data for 785 patients.

**Fig. 1:**
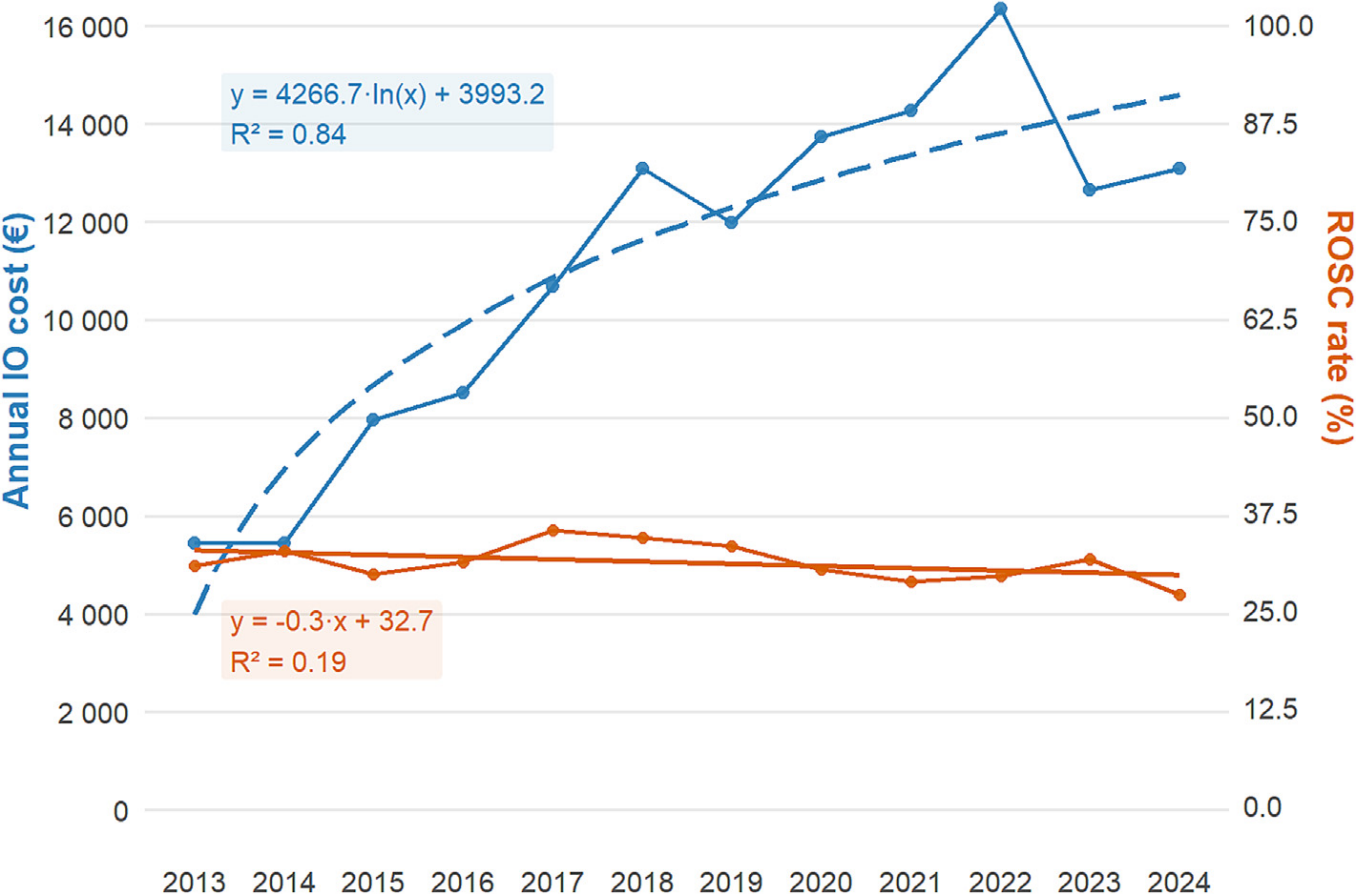
**Temporal trends in intraosseous access costs and ROSC rates, 2013–2024**. IO, intraosseous; IV, peripheral intravenous; ROSC, return of spontaneous circulation. Dashed line: IO cost (log fit); solid line: ROSC rate (linear fit).

The cost of IO access increased logarithmically over the study period (*R*^2^ = 0.84) (Fig. 1). It was lowest in 2013 at €5450 and peaked in 2022 at €16,350. Over the period 2013–2024, the cumulative cost of IV access was €2555, compared with €133,198 for IO access. ROSC was obtained in 1651 (31%) patients, with a slight decreasing trend over time (−0.3% per year, *R*^2^ = 0.19), while 30-day survival was obtained in 151 (2.8%) patients (−0.08% per year; *R*^2^ = 0.10). The median annual number of OHCAs having received adrenaline was 438 (IQR: 423–462), the median annual number of ROSC was 136 (IQR: 121–144), and the median annual number of 30-day survivors was 12 (IQR: 9–14).

**Fig. 2:**
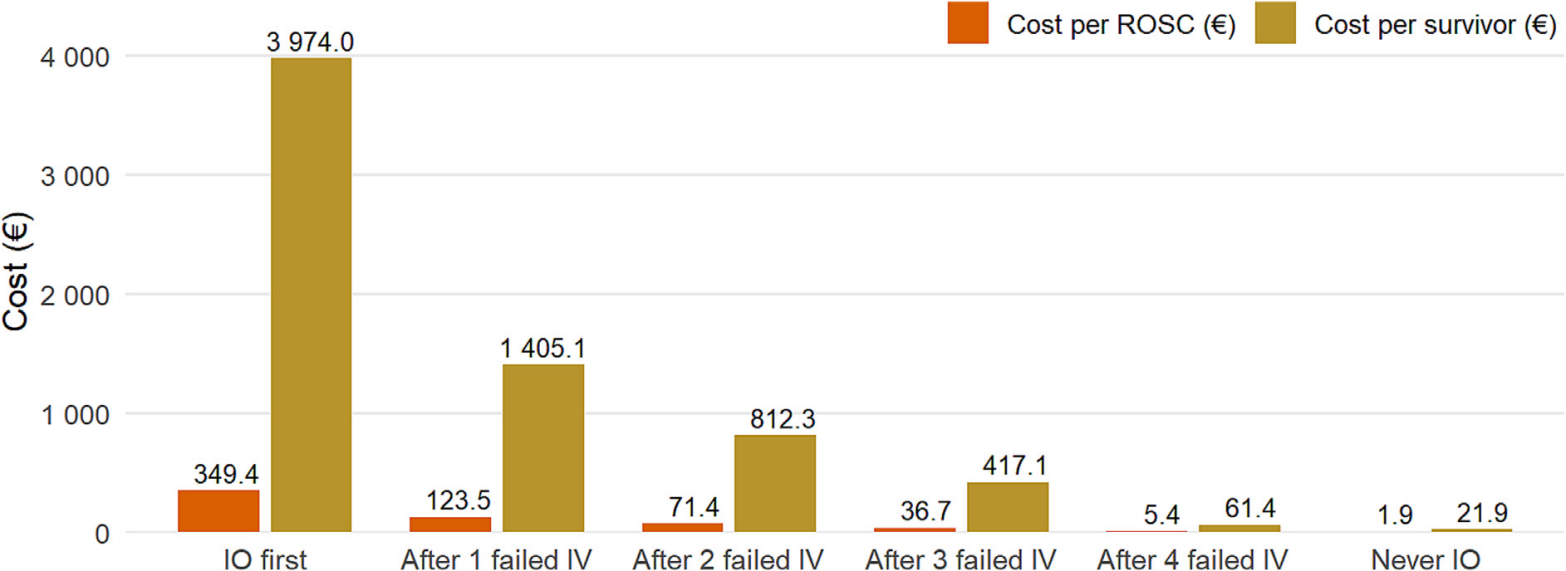
**Scenario-based cost of vascular access per outcome**. IO, intraosseous; IV, peripheral intravenous; ROSC, return of spontaneous circulation. Modeling assumed cumulative IV success of 65%, 80%, 90%, and 99% after 1, 2, 3, and 4 IV attempts, respectively. IO was assumed to succeed at first attempt (100%). “Never IO” indicates that no IO cost was assigned.

Scenario-based modeling applied to the cohort’s median annual values showed that a systematic first-line IO strategy would be associated with an annual cost of €47,688, corresponding to €349 per ROSC and €3974 per survivor (Table 2). Delaying IO after failed IV attempts substantially reduced costs: €16,861 after one failed attempt, €9747 after two, €5005 after three, and €737 after four. In the strategy without IO, the annual cost was €262, corresponding to €2 per ROSC and €22 per survivor (Fig. 2).

**Table 2:** **Scenario-based cost-minimization of intraosseous and intravenous access.** IO, intraosseous; IV, peripheral intravenous; ROSC, return of spontaneous circulation. Modeling assumed cumulative IV success of 65%, 80%, 90%, and 99% after 1, 2, 3, and 4 IV attempts, respectively. IO was assumed to succeed at first attempt (100%). “Never IO” indicates that no IO cost was assigned.

**Scenario**	**IV cost (€)**	**IO cost (€)**	**Total cost (€)**	**Cost per** **ROSC (€)**	**Cost per survivor (€)**
IO first	0.0	47,687.5	47,687.5	349.4	3974.0
IO after 1 failed IV	170.6	16,690.6	16,861.2	123.5	1405.1
IO after 2 failed IV	210.0	9537.5	9747.5	71.4	812.3
IO after 3 failed IV	236.2	4768.7	5005.0	36.7	417.1
IO after 4 failed IV	259.9	476.9	736.8	5.4	61.4
Never IO	262.5	0.0	262.5	1.9	21.9

## Discussion

In our population-based cohort, IO access accounted for 20% of vascular access attempts and generated more than €130,000 in device-related costs over the study period. IO-first strategies were associated with estimated device costs of €349 per ROSC and €3974 per survivor, compared with €2 and €22 respectively for a strategy without IO. These estimates assumed 100% IO success and excluded the driver cost; incorporating a lower success rate or device amortization would further worsen the costs.^11^ Overall outcomes (ROSC and 30-day survival) remained stable over time despite the increasing use of IO access. While IO remains important as a rescue option when IV access is unachievable, our findings suggest that systematic first-line use of IO is more costly and not supported by current clinical evidence in the general OHCA population requiring adrenaline.

To our knowledge, no published study has specifically assessed the economic implications of IO versus IV access in OHCA. Although the PARAMEDIC-3 trial protocol includes a planned health economic evaluation, results have not yet been reported.^12^ The necessity of a dedicated cost-focused analysis is underscored by the fact that recent RCTs comparing IO-first vs IV-first found no clear benefit for IO access.^5^, ^6^ In a 2026 meta-analysis restricted to PARAMEDIC-3 and IVIO trials (7561 patients), an IO-first strategy was not associated with improved 30-day survival (OR 0.97, 95% CI 0.80–1.18) or favorable neurological outcome (OR 1.03, 95% CI 0.81–1.31), did not increase ROSC at any time (OR 0.91, 95% CI 0.83–1.00), and was associated with lower odds of sustained ROSC (OR 0.89, 95% CI 0.80–0.99).^13^

Our findings echo prior economic evaluations of high-cost resuscitation technologies. The cost-effectiveness study of the mechanical chest compression device LUCAS-2, based on trial data, found that manual CPR was less costly and produced better or equal outcomes in most analyses, including lifetime extrapolations using QALYs.^14^, ^15^, ^16^ These observations illustrate a recurrent theme in resuscitation research: many technological innovations are expensive yet yield limited or uncertain incremental benefit.^17^ From a health-systems perspective, our results underscore the need to evaluate both clinical efficacy and economic sustainability of prehospital interventions, particularly in resource-constrained settings.^18^, ^19^ Widespread adoption of costly devices or strategies without demonstrable benefit may divert resources from established, cost-effective measures, such as bystander CPR training and public access defibrillation.^20^, ^21^

We must acknowledge several limitations inherent in registry-based analyses. Confounding by indication is unavoidable: IO is more likely to be deployed in cases where IV access is difficult or has already failed, which selects patients at higher risk of poor outcomes. Therefore, the numerically lower observed outcomes associated with IO should not be construed as proof of inferiority in this study. The register does not record the number of IV attempts, procedural context, or operator-level factors such as experience. It also does not capture whether multiple IO punctures were required, which likely occurs in practice and would further increase the true economic burden of IO use. We assumed 100% IO success for scenario modeling. However, in the IVIO trial, first successful vascular access according to the assigned IO strategy was 90%, indicating that this simplifying assumption likely favored IO and may have underestimated the excess cost of IO-first strategies. Finally, results should be interpreted as descriptive, reflecting population-level patterns rather than causal effects.

## Conclusions

In this cost-minimization analysis, IO use increased over time and generated substantially higher device-related costs. As RCTs have not demonstrated clinical superiority of IO over IV access in OHCA, an IV-first strategy appears economically preferable, reserving IO access as a fallback option when IV attempts are unsuccessful.

## Data sharing statement

The datasets used and/or analyzed during the current study are available from the corresponding author upon reasonable request.

## Funding statement

This research did not receive any specific grant from funding agencies in the public, commercial, or not-for-profit sectors.

## CRediT authorship contribution statement

**Alexis Marouk:** Writing – review & editing, Writing – original draft, Visualization, Methodology, Formal analysis, Conceptualization. **Jean-Marc Agostinucci:** Writing – review & editing, Writing – original draft, Visualization, Supervision, Project administration, Methodology, Investigation, Data curation, Conceptualization. **François-Pierre Auffredou:** Writing – review & editing, Investigation. **Tomislav Petrovic:** Writing – review & editing, Investigation. **Jacques Metzger:** Writing – review & editing, Investigation. **Philippe Bertrand:** Writing – review & editing, Investigation. **Priscilia Hsing:** Writing – review & editing, Investigation. **Anne-Laure Feral-Pierssens:** Writing – review & editing, Validation, Supervision. **Frédéric Lapostolle:** Writing – review & editing, Writing – original draft, Visualization, Validation, Supervision, Project administration, Methodology, Formal analysis, Conceptualization.

## Declaration of competing interest

The authors declare that they have no conflicts of interest to disclose.
